# Association of Daily Steps with Incident Nonalcoholic Fatty Liver Disease: Evidence from the UK Biobank Cohort

**DOI:** 10.1249/MSS.0000000000003738

**Published:** 2025-04-24

**Authors:** EVELYNNE S. FULDA, LAURA PORTAS, CHARLIE HARPER, DAVID PREISS, DERRICK BENNETT, AIDEN DOHERTY

**Affiliations:** 1 Big Data Institute, Nuffield Department of Population Health, University of Oxford, Oxford, UNITED KINGDOM; 2 Clinical Trial Service Unit & Epidemiological Studies Unit, Nuffield Department of Population Health, University of Oxford, Oxford, UNITED KINGDOM

**Keywords:** EXERCISE, WALKING, ACCELEROMETRY, PROSPECTIVE STUDIES

## Abstract

**Purpose:**

Low physical activity has been shown to be associated with a higher risk of nonalcoholic fatty liver disease (NAFLD). However, the strength and shape of this association are currently uncertain due to a reliance on self-reported physical activity measures. This report aims to investigate the relationship of median daily step count with NAFLD using accelerometer-derived step count from a large prospective cohort study.

**Methods:**

The wrist-worn accelerometer substudy of the UK Biobank (*N* = ~100,000) was used to characterize median daily step count over a 7-d period. NAFLD cases were ascertained via record linkage with hospital inpatient data and death registers or by using a measure of liver fat from imaging. Cox proportional hazards models were employed to assess the association between step count and NAFLD, adjusting for age, sociodemographic, and lifestyle factors. Mediation analyses were conducted.

**Results:**

Among 91,031 participants (709,440 person-years of follow-up), there were 762 incident NAFLD cases. Higher step count was log-linearly and inversely associated with risk of NAFLD. A 1000-step increase (representing 10 min of walking) was associated with a 12% (95% confidence interval, 10%–14%) lower hazard of NAFLD. When using imaging to identify NAFLD, a 1000-step increase was associated with a 6% (95% confidence interval, 6%–7%) lower risk. There was evidence for mediation by adiposity, accounting for 39% of the observed association.

**Conclusions:**

Daily step count, a modifiable risk factor, is log-linearly and inversely associated with NAFLD. This association was only partially explained by adiposity. These findings from a large cohort study may have important implications for strategies to lower NAFLD risk.

Nonalcoholic fatty liver disease (NAFLD) is the most common cause of chronic liver disease, with an estimated global prevalence of 30% (1). It is associated with multiple comorbidities, is a leading cause of cirrhosis and liver failure, and is predicted to be the most common indication necessitating liver transplantation over the next decade (2). Importantly, NAFLD prevalence is increasing, primarily as a result of population-wide lifestyle changes (3). Thus, lifestyle modifications, such as changes in physical activity, may represent an opportunity for primary prevention of NAFLD. For example, prospective studies have suggested that higher levels of physical activity are associated with a lower risk of incident NAFLD (4–13), and clinical trials have demonstrated evidence for resolution of fatty liver with increased exercise (14).

However, the current physical activity and NAFLD evidence bases have several limitations. First, most previous studies have used subjective measures of activity. These measures are prone to substantial measurement error and bias, do not measure activity continuously, are difficult to compare between subjects, and only capture intentional physical activity (15). In addition, physical activity may be characterized in unintuitive ways or as relative measures of activity, which are population-dependent. Finally, there is limited understanding of how adiposity, hypertension, medication use, or diabetes potentially mediate the association between physical activity and NAFLD risk. Prior studies have tended to treat these covariates as only confounders, without considering their intermediary role (16).

Using comprehensive data from the UK Biobank, we aimed to 1) investigate the association between accelerometer-measured physical activity characterized as median daily step count and incident NAFLD, and 2) consider how potential factors mediate this association. In pursuing these aims, this work builds on prior studies in the UK Biobank investigating physical activity and NAFLD associations (5,9) by addressing these important research gaps.

## METHODS

### Study design and participants

The UK Biobank is a prospective cohort study that enrolled ~500,000 adults between 2006 and 2010 across England, Scotland, and Wales (17). Full information on the UK Biobank study design, recruitment strategy, and data collection protocols has been previously described (17). Briefly, participants were eligible to take part in the study if they were aged 40–69 yr and resided within driving distance of an assessment center at the time of recruitment. All participants provided written informed consent and completed a baseline visit involving collection of sociodemographic, lifestyle, and health information. Excepting cases of withdrawal of consent, migration, and/or death, participants have been passively followed continuously since enrollment. This research conforms to the ethical guidelines of the Declarations of Helsinki and Istanbul and was approved by the Northwest Multi-centre Research Ethics Committee as a Research Tissue Bank.

To derive the main analysis cohort (Supplemental Fig. 1, Supplemental Digital Content, http://links.lww.com/MSS/D235), participants without accelerometer data or with accelerometer data not meeting quality standards, prevalent liver disease (NAFLD and other liver diseases assessed using record linkage; *International Classification of Diseases* (*ICD*) codes outlined in Supplemental Table 1, Supplemental Digital Content, http://links.lww.com/MSS/D235) or history of alcohol misuse (assessed using record linkage; *ICD* codes in Supplemental Table 1, Supplemental Digital Content, http://links.lww.com/MSS/D235) at the time of accelerometer wear, or missing covariate data were excluded.

### Physical activity assessment

A random selection of participants with a valid email address (*N* = 236,519) was invited to wear a wrist-worn accelerometer for 7 d (18). Forty-five percent of participants volunteered, with data collection commencing in June 2013 and concluding in December 2015 (18). During this period, participants were mailed an Axivity AX3 triaxial accelerometer that was programmed to automatically capture data 2 d after postage at 10:00 am and to measure continuously. Participants were instructed to wear the accelerometer without interruption on their dominant wrist and to conduct their normal activities.

From the raw accelerometer data, daily step count was quantified using a hybrid self-supervised machine learning and peak detection algorithm validated against reference video measurements from personal wearable cameras (19). The median daily step count over the wear period was obtained. Step count was categorized into quartiles to look at the overall shape of the association, with the first (lowest) quartile (0–6999 steps) used as the reference group to allow for investigation of a dose–response relationship of increasing step counts. As the shape was log-linear, continuous step count was also assessed. A cadence (walking pace) of 100 steps per minute is considered moderate (20); thus, the continuous step count variable is presented as per 1000-step increase, representing on average 10 more minutes of walking per day.

Physical activity was additionally assessed using the International Physical Activity Questionnaire (IPAQ). This questionnaire assessed how many days per week a participant completed more than 10 min each of walking, moderate-level activity, or vigorous-level activity, as well as how many minutes per day a participant typically spends on each activity type based on self-report. Cassidy et al. (21) processed these responses to derive total metabolic equivalent of task (MET)-minutes per week for all activity according to IPAQ guidelines (22).

### NAFLD ascertainment

NAFLD cases were identified through linkage to routinely collected hospital admission and national death registry data using *ICD-10* (K75.8 and K76.0) and *ICD-9* (571.5, 571.8, and 571.9) codes. This analysis linked to both primary and secondary *ICD* codes to capture participants admitted both for and with NAFLD, and to capture NAFLD as either an underlying or contributory cause of death.

A subset of UK Biobank participants underwent magnetic resonance imaging (MRI) with subsequent processing of imaging-derived phenotypes, with methods as described by Wilman et al. (23). To assess how the NAFLD ascertainment method may affect findings, among participants from the main analysis with both accelerometer and imaging data, NAFLD was defined using proton density fat fraction (PDFF) derived from MRI of the liver. A PDFF >5.5% is considered to be indicative of NAFLD (23). Imaging of participants commenced on April 30, 2014.

Participants were censored at the time of their first NAFLD diagnosis, death, withdrawal from the study, or end of follow-up. Record linkage censoring dates varied by region: October 31, 2022, for participants in England; August 31, 2022 for participants in Scotland; and May 31, 2022, for participants in Wales.

### Covariates

At the baseline visit, information relating to the following covariates was collected (Supplemental Table 2, Supplemental Digital Content, http://links.lww.com/MSS/D235): sex assigned at birth, self-reported race/ethnicity (grouped as White and non-White due to limited racial and ethnic diversity in the cohort), Townsend deprivation index, educational attainment, weekly alcohol intake frequency, smoking status, fruit/vegetable intake, self-rated health, body mass index (BMI), systolic blood pressure, medication use, number of unique hospital episodes, and personal medical history for cancer, diabetes, hypertension, and chronic lower respiratory disease. For covariates that allowed participants to respond with “I don’t know” or “Prefer not to answer,” these responses were interpreted as missing.

### Statistical analysis

Cox proportional hazards regression models using age as the timescale were employed to assess the association between device-measured step count and risk of NAFLD. The model was adjusted for sex at birth, race/ethnicity, Townsend deprivation index, educational attainment, alcohol consumption, smoking status, and fruit/vegetable consumption (adjusted model). Hazard ratios (HRs) are presented with their accompanying 95% confidence intervals (CI) using floating absolute risks where appropriate to present group-specific risks and their variances (24). Analyses were repeated using MET-minutes per week as the exposure.

Mediation analyses were conducted for covariates thought to lie on the mechanistic pathway between step count and NAFLD. The included variables were BMI, hypertension, medication use, and diabetes. The effect of mediation was assessed in three ways: reduction in *χ*^2^, calculation of risk mediated, and causal mediation. To investigate the reduction in *χ*^2^, a likelihood ratio test was performed by fitting the adjusted model with and without step count (model A). Next, a likelihood ratio test was performed by fitting the adjusted model with the addition of the mediator(s) with and without step count (model B). Finally, the reduction in the likelihood ratio *χ*^2^ test statistic from model A to model B was assessed. Percent of risk mediated (PRM) was estimated as per the Global Burden of Metabolic Risk Factors for Chronic Diseases Collaboration (16):


$$\mathrm{PRM}=\frac{{\mathrm{HR}}_{\left(\text{confounder adjusted}\right)}-{\mathrm{HR}}_{\left(\text{confounder and mediator adjusted}\right)}}{{\mathrm{HR}}_{\left(\text{confounder adjusted}\right)}-1}\times 100$$

Each mediator was assessed individually, in all combinations of two, and all together (16). Finally, a formal causal mediation analysis was conducted for each mediator individually using the *mediate* package in Stata. The total effect (the difference in NAFLD risk if everyone exercised like those in the most active quarter compared with the least) was quantified and split into the natural direct effect (the effect of the exposure on the outcome independent of the mediator) and the natural indirect effect (the effect of the exposure on the outcome due to the mediator). We report the proportion mediated, which is calculated as:


$$\text{Proportion mediated}=\frac{\text{Natural Indirect Effect}}{\text{Natural Indirect Effect}+\text{Natural Direct Effect}}$$

This method involves a counterfactual framework, as described elsewhere (25).

Four sensitivity analyses were conducted. The primary analysis relies on record linkage; therefore, the observed association may be due to physical activity being associated with risk of hospitalization or death. To account for this potential ascertainment bias, the first sensitivity analysis excluded participants with self-reported poor health or potentially relevant diseases (cancer, chronic lower respiratory disease, diabetes, and hypertension) and additionally adjusted for the unique number of hospital episodes. The second sensitivity analysis excluded participants with events in the first 5 yr of follow-up to consider the potential for reverse causation. For the third sensitivity analysis, models from the main analysis were repeated using either PDFF-defined NAFLD or record linkage-defined NAFLD among participants with both accelerometer and imaging data. Participants with prevalent NAFLD based on PDFF were additionally excluded. As imaging visits were often conducted after accelerometer wear, it was not possible to exclude all prevalent PDFF-defined NAFLD cases. Therefore, to allow for a cross-sectional analysis, the relationship between step count and log-transformed PDFF (transformed due to skew of distribution) was assessed using multivariable linear regression. The final sensitivity analysis sought to investigate the potential for unmeasured and residual confounding by calculating the *E*-value for the main analysis as follows (26):


$$E-\text{value}=\mathrm{RR}+\sqrt{\mathrm{RR}\times \left(\mathrm{RR}-1\right)}$$

where RR is the observed risk ratio. For risk ratios <1, the inverse of the risk ratio is used. The *E*-value quantifies the necessary strength of association for an unmeasured or inadequately measured confounder to explain away the association between the exposure and outcome, after adjustment for measured confounders (26):

To determine the number of events needed to detect a meaningful association, we conducted a literature review and meta-analysis for prospective associations between physical activity and NAFLD. Methods are described in detail in the supplemental methods (Supplemental Digital Content, http://links.lww.com/MSS/D235). Briefly, there were 10 publications (4–13) included in the literature review and meta-analysis (Supplemental Figs. 2 and 3, Supplemental Tables 3, 4, and 5, Supplemental Digital Content, http://links.lww.com/MSS/D235). All studies demonstrated a significant inverse association between physical activity and risk of NAFLD. The overall fixed-effects pooled HR when comparing the most active to the least active was 0.82 (95% CI, 0.81–0.84) with very considerable heterogeneity (*I*^2^ = 93%; *P* < 0.01; Supplemental Figure 3, Supplemental Digital Content, http://links.lww.com/MSS/D235). Therefore, at a power of 0.80 and an alpha level of 0.05, 798 events would be needed to detect an HR of 0.82 (27).

Normally distributed continuous variables are presented as mean (standard deviation (SD)), non-normally distributed continuous variables are presented as median (interquartile range (IQR)), and categorical variables are presented as *n* (%). A two-sided alpha level of 0.05 was considered statistically significant.

Data were extracted from the UK Biobank Research Analysis platform using pyspark, with methods as previously described (18). Data were preprocessed using R, version 4.3.1 (R Core Team, Vienna, Austria). All statistical analyses were conducted using Stata, version 18.0 (StataCorp, College Station, TX). Results are reported according to the STROBE guidelines (Supplemental Table 3, Supplemental Digital Content, http://links.lww.com/MSS/D235).

### NAFLD and metabolic-associated steatotic liver disease

A recent Delphi consensus statement has provided new nomenclature to replace NAFLD with metabolic dysfunction–associated steatotic liver disease (MASLD) (28). Our study uses the term NAFLD instead of MASLD as 1) currently, there are no MASLD-specific *ICD* codes, and therefore, diagnosis using record linkage poses a challenge; 2) an MASLD diagnosis encompasses the presence of both steatosis and metabolic dysfunction and, in our analysis, we wanted to investigate the unique combinations of NAFLD with different metabolic complications separately; 3) NAFLD diagnosis relies on linkage with hospital admission and death data in this analysis, and accompanying metabolic data (e.g., systolic blood pressure, BMI, etc.) may not be available at the same time point, which may lead to missing MASLD cases; and 4) we were interested in comparing results from our analysis to prior studies, all of which used the NAFLD diagnostic definition.

## RESULTS

### Baseline characteristics

After excluding 12,328 participants, 91,031 participants were included in the present analysis (flow diagram in Supplemental Fig. 1, Supplemental Digital Content, http://links.lww.com/MSS/D235). The median age of participants was 63.4 (IQR, 56.2–68.5) yr, 88,320 (97%) participants were White, and 51,647 (57%) participants were female (Table 1). Compared with participants in the first (lowest) step count quarter, participants in the fourth (highest) step count quarter were more likely to be younger (62.7 (IQR, 56.0–67.8) vs 64.0 (IQR, 56.6–69.3) yr), consume alcohol more frequently (54% drank ≥3 times a week compared with 42%), and have lower BMI (25.6 (SD, 3.7) vs 28.2 (SD, 5.4) kg·m^−2^).

**TABLE 1:** Baseline participant characteristics, overall and by median daily step count quarter.

	Quarter 1: 0–6999 Steps (*N* = 22,764)	Quarter 2: 7000–9269 Steps (*N* = 22,753)	Quarter 3: 9270–11,891 Steps (*N* = 22,760)	Quarter 4: 11,892–41,189 Steps (*N* = 22,754)	Total: 0–41,189 Steps (*N* = 91,031)
Median daily step count	5259 (1393)	8150 (647)	10490 (749)	14783 (2763)	9670 (3845)
MET·min·wk^−1^	1333 (570–2652)	1635 (796–3066)	1857 (946–3416)	2319 (1235–4158)	1773.0 (857–3339)
Age at entry, yr	64.0 (56.6–69.3)	63.3 (56.0–68.6)	63.4 (56.2–68.4)	62.7 (56.0–67.8)	63.4 (56.2–68.5)
Female sex	13,067 (57.4%)	13,129 (57.7%)	12,934 (56.8%)	12,517 (55.0%)	51,647 (56.7%)
White race/ethnicity	22,011 (96.7%)	22,058 (96.9%)	22,124 (97.2%)	22,127 (97.2%)	88,320 (97.0%)
Townsend deprivation index quintile					
Least deprived	11,132 (48.9%)	11,992 (52.7%)	11,726 (51.5%)	11,410 (50.1%)	46,260 (50.8%)
Second quarter	6623 (29.1%)	6447 (28.3%)	6614 (29.1%)	6531 (28.7%)	26,215 (28.8%)
Third quarter	1782 (7.8%)	1653 (7.3%)	1687 (7.4%)	1857 (8.2%)	6979 (7.7%)
Fourth quarter	2362 (10.4%)	2024 (8.9%)	2047 (9.0%)	2224 (9.8%)	8657 (9.5%)
Most deprived	865 (3.8%)	637 (2.8%)	686 (3.0%)	732 (3.2%)	2920 (3.2%)
Highest education					
School leaver	11,129 (48.9%)	9918 (43.6%)	9159 (40.2%)	8763 (38.5%)	38,969 (42.8%)
Further education	3139 (13.8%)	3079 (13.5%)	2897 (12.7%)	2980 (13.1%)	12,095 (13.3%)
Higher education	8496 (37.3%)	9756 (42.9%)	10,704 (47.0%)	11,011 (48.4%)	39,967 (43.9%)
Alcohol consumption					
3+ times a week	9475 (41.6%)	11,003 (48.4%)	11,791 (51.8%)	12,289 (54.0%)	44,558 (48.9%)
<3 times a week	11,673 (51.3%)	10,550 (46.4%)	9848 (43.3%)	9369 (41.2%)	41,440 (45.5%)
Never	1616 (7.1%)	1200 (5.3%)	1121 (4.9%)	1096 (4.8%)	5033 (5.5%)
Never smoker	12,400 (54.5%)	13,223 (58.1%)	13,453 (59.1%)	13,435 (59.0%)	52,511 (57.7%)
Fruit and vegetable consumption					
<3 servings a day	1114 (4.9%)	727 (3.2%)	642 (2.8%)	625 (2.7%)	3108 (3.4%)
3–4.9 servings a day	3328 (14.6%)	3000 (13.2%)	2774 (12.2%)	2506 (11.0%)	11,608 (12.8%)
5–7.9 servings a day	8322 (36.6%)	8440 (37.1%)	8352 (36.7%)	8183 (36.0%)	33,297 (36.6%)
>8 servings a day	10,000 (43.9%)	10,586 (46.5%)	10,992 (48.3%)	11,440 (50.3%)	43,018 (47.3%)
Self-reported health					
Poor	1137 (5.0%)	451 (2.0%)	321 (1.4%)	231 (1.0%)	2140 (2.4%)
Fair	4841 (21.3%)	3391 (14.9%)	2976 (13.1%)	2623 (11.5%)	13,831 (15.2%)
Good	13,061 (57.4%)	13,853 (60.9%)	13,955 (61.3%)	13,982 (61.4%)	54,851 (60.3%)
Excellent	3725 (16.4%)	5058 (22.2%)	5508 (24.2%)	5918 (26.0%)	20,209 (22.2%)
BMI, kg·m^−2^	28.2 (5.4)	26.7 (4.3)	26.1 (4.0)	25.6 (3.7)	26.7 (4.5)
Medication use*^a^*	6629 (29.1%)	5094 (22.4%)	4741 (20.8%)	4003 (17.6%)	20,467 (22.5%)
Diabetes	1165 (5.1%)	601 (2.6%)	436 (1.9%)	343 (1.5%)	2545 (2.8%)
Hypertension	4212 (18.5%)	2929 (12.9%)	2546 (11.2%)	2115 (9.3%)	11,802 (13.0%)

Data are presented as mean (standard deviation) for normally distributed continuous measures, as median (IQR) for non-normally distributed continuous measures, and *n* (%) for categorical measures.

*^a^* Use of cholesterol-lowering medication, blood pressure medication, and/or insulin.

### Associations between daily step count and incident NAFLD

**FIGURE 1:**
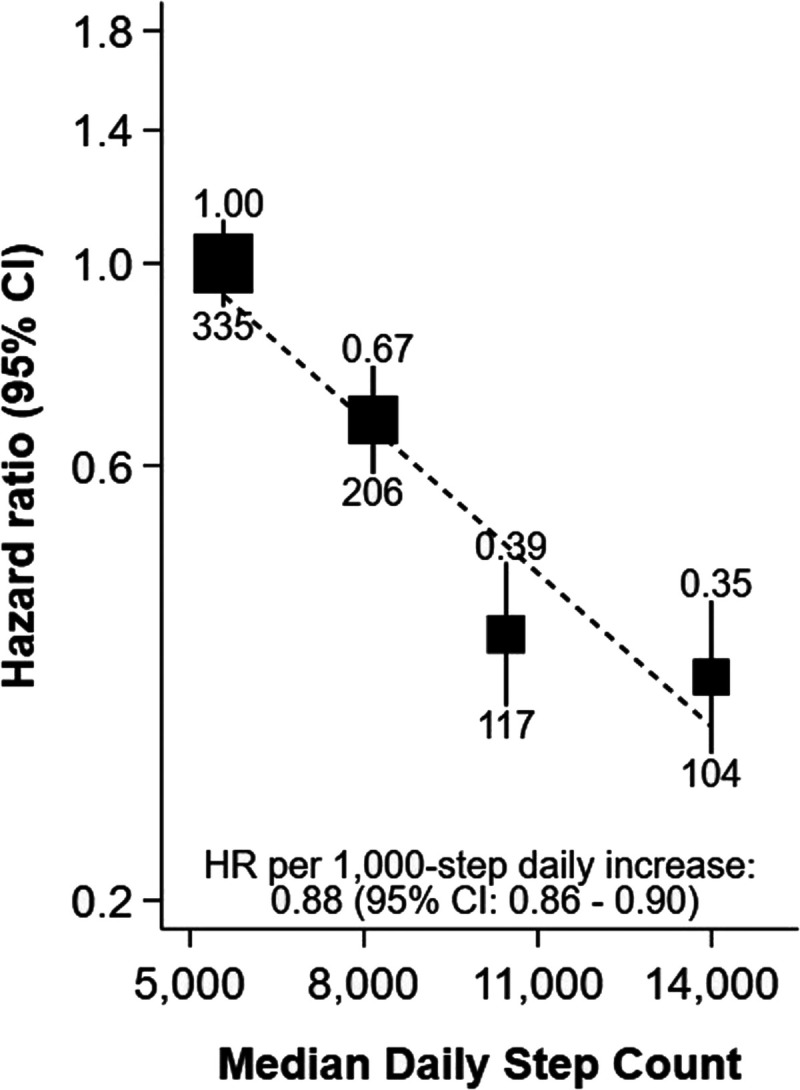
Association of quartiles of accelerometer-measured daily step count with risk of NAFLD after a median of 7.9 yr of follow-up in 91,031 UK Biobank participants. Adjusted for sex at birth, race/ethnicity, Townsend deprivation index, educational attainment, alcohol consumption, smoking status, fruit and vegetable consumption, and using age as the time scale; the number above each vertical line is the HR, and the number below each vertical line is the number of events; the dotted line shows the log-linear model.

Over a median follow-up time of 7.9 (IQR, 7.3–8.4) yr (709,440 person-years), there were 762 incident NAFLD cases identified using record linkage. Only 43 participants were admitted with NAFLD as the primary hospital diagnosis. The next most common primary diagnoses were R10.1 (pain localized to upper abdomen; *n* = 21) and K80.1 (calculus of gallbladder with other cholecystitis; *n* = 21). Furthermore, only one participant had NAFLD listed as their primary death cause. After adjustment for measured confounders, compared with participants in the first step count quarter, participants in the fourth step count quarter had an HR of 0.35 (95% CI, 0.29–0.43; likelihood ratio test for trend, *χ*^2^ = 129, *P* < 0.001; Fig. 1). A 1000-step higher step count (10 min of walking) was associated with a 12% lower hazard of NAFLD (0.88, 0.86–0.90). Sequential adjustment of confounders is shown in Supplemental Figure 4 (Supplemental Digital Content, http://links.lww.com/MSS/D235). When using self-report to characterize physical activity, participants in the fourth MET-minutes per week quarter had a 39% lower hazard of NAFLD than those in the first (0.61, 0.51–0.72; Supplemental Fig. 5, Supplemental Digital Content, http://links.lww.com/MSS/D235).

### Mediation analysis

**FIGURE 2:**
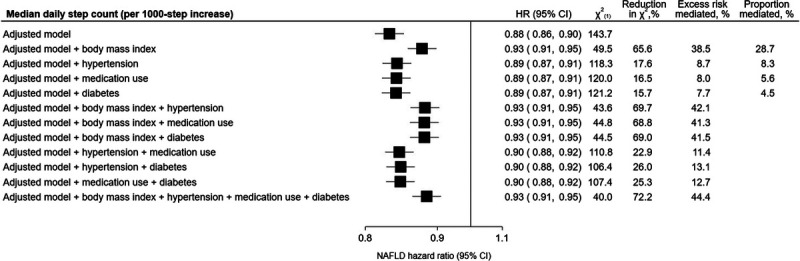
Association of accelerometer-measured daily step count with risk of NAFLD and inclusion of potential mediators. Adjusted model: controlled for sex at birth, race/ethnicity, alcohol consumption, smoking status, educational attainment, Townsend deprivation index, fruit and vegetable consumption, and using age as the time scale. Reduction in *χ*^2^ (in relation to adjusted model), quantifies the amount the observed association is due to the mediator(s). Risk mediated, quantifies the percent change in the effect estimate after addition of the mediator(s). Proportion mediated, quantifies the proportion of the total effect that is due to the mediator.

Adding BMI into the model noticeably attenuated the association between step count and risk of NAFLD (0.93, 0.91–0.95), with a reduction in the likelihood ratio test statistic of 66% and a PRM of 39% (Fig. 2). Adding hypertension, medication use, or diabetes into the model also attenuated the association, although with a smaller effect (hypertension: 0.89, 0.87–0.91, reduction in *χ*^2^: 18%, PRM: 9%; medication use: 0.89, 0.87–0.91, reduction in *χ*^2^: 17%, PRM: 8%; diabetes: 0.89, 0.87–0.91, reduction in *χ*^2^: 16%, PRM: 8%; Fig. 2). In models including multiple mediators, the attenuation of the HR, reduction in *χ*^2^, and PRM was largely driven by the inclusion of BMI. Furthermore, in causal mediation models, BMI showed the highest proportion of mediation (29%; Fig. 2). When restricting analyses to only include participants with obesity (BMI ≥30 kg·m^−2^), the association between step count and hazard of NAFLD was attenuated (0.92, 0.89–0.95) and no longer showed a clear log-linear dose–response pattern (Supplemental Fig. 6, Supplemental Digital Content, http://links.lww.com/MSS/D235).

### Sensitivity analyses

Exclusion of events that occurred during the first 5 yr of follow-up (389 events) or exclusion of individuals with ill health and controlling for number of hospitalizations (*n* = 26,580) both marginally attenuated the association between step count and hazard of NAFLD: HR per 1000-step increase (95% CI): 0.90 (0.87–0.92) and 0.90 (0.88–0.93), respectively (Supplemental Fig. 7, Supplemental Digital Content, http://links.lww.com/MSS/D235).

**FIGURE 3:**
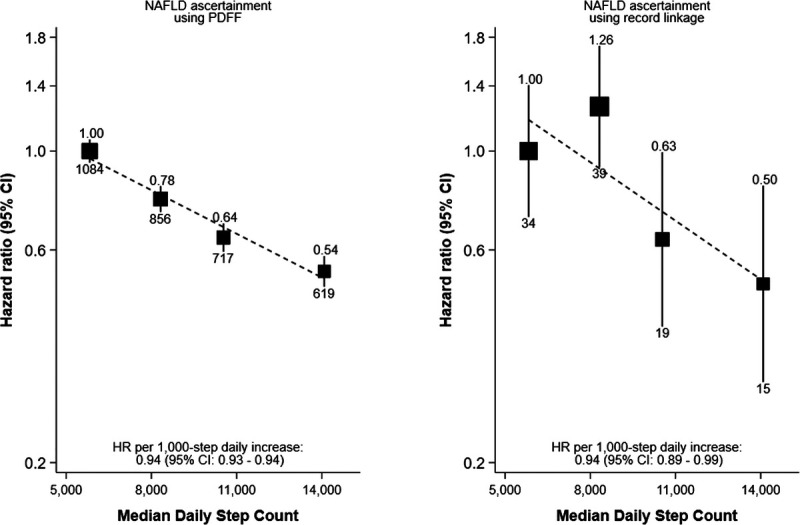
Association of quartiles of accelerometer-measured daily step count with risk of NAFLD in 15,689 UK Biobank participants with both accelerometer and imaging data. Adjusted for sex at birth, race/ethnicity, Townsend deprivation index, educational attainment, alcohol consumption, smoking status, fruit and vegetable consumption, and using age as the time scale; the number above each vertical line is the HR, and the number below each vertical line is the number of events; the dotted line shows the log-linear model.

Among participants with accelerometer and imaging data (*N* = 15,689), there were 3276 NAFLD cases based on PDFF and 107 NAFLD cases based on record linkage, with median follow-up times of 7.6 (IQR, 6.9–8.1) and 7.8 (IQR, 7.4–8.3) yr, respectively. The association between step count and risk of NAFLD was attenuated compared with the main analysis for both PDFF-ascertained NAFLD (0.94, 0.93–0.94) and record linkage-ascertained NAFLD (0.94, 0.89–0.99) (Fig. 3). Step count was inversely associated with PDFF in linear regression analyses: each 1000-step increase in step count was associated with a 2% lower PDFF value on average (Supplemental Fig. 8, Supplemental Digital Content, http://links.lww.com/MSS/D235).

The *E*-value for the main analysis HR of 0.88 (0.86–0.90) was 1.52 with an interval bound of 1.46. This means that, after adjustment for other measured confounders, an unmeasured or inadequately measured confounder would need to be associated with both step count and NAFLD risk by an HR of 1.52 to explain away the observed association, or an HR of 1.46 for the observed association CI to include the null value. This represents a relatively large necessary association, as all included confounders had an HR below this threshold, excepting alcohol consumption.

## DISCUSSION

To the best of our knowledge, this is the largest study of device-measured daily step count and risk of NAFLD, yielding three key findings. First, higher median daily step count was associated with a lower risk of NAFLD, and this association is much stronger than has been previously reported (4–13). Second, this association was independent, but partially mediated by BMI, hypertension, medication use, and diabetes. Finally, the observed association was consistent, but attenuated, when considering MRI-based NAFLD assessments.

A striking result from the present analysis is a much stronger than previously reported steady decline in NAFLD risk with increasing daily step count in tandem with evidence of a log-linear dose–response relationship. Although prior prospective studies have demonstrated this association, our study shows at least double the strength of the association. A literature review and meta-analysis of prior studies undertaken as part of this analysis demonstrated a fixed-effects pooled HR of 0.82 (0.81–0.84; *I*^2^ = 93%) when comparing those who are most physically active to those who are least physically active. For comparison, our study demonstrated an HR of 0.35 (0.29–0.43), comparing those in the highest step count quarter to those in the lowest. This much stronger association may be explained by the use of objective physical activity measurements, as 8 of the 10 studies in the meta-analysis utilized questionnaire-based instruments to characterize activity (4–8,10–12). Indeed, a recent meta-analysis conducted by Ekelund and colleagues (29) demonstrated that effect sizes for associations between physical activity and mortality were twice as large for accelerometer-measured physical activity compared with self-reported physical activity. This likely reflects that objective instruments provide more valid, reliable, and comprehensive activity estimates (15). Indeed, in our analysis, the observed activity-NAFLD effect size was halved when using a self-reported physical activity measurement compared with an accelerometer measurement. However, it should be noted that self-reported MET-min/week are not directly comparable to accelerometer-measured step counts and there was a time gap between IPAQ assessment and accelerometer assessment.

Median daily step count was independently associated with NAFLD risk; however, there were several relevant mediating factors, principally, obesity. One hypothesis for the observed association between median daily step count and risk of NAFLD is that lower levels of activity lead to higher levels of adiposity, specifically in the form of visceral adipose tissue, leading to infiltration of fat into hepatocytes driven by free fatty acid efflux from adipose tissue (6). The present analysis demonstrated that adding BMI to the model attenuated the association between step count and risk of NAFLD, providing evidence to support this hypothesis. Other measures of central adiposity (body fat percentage, waist circumference) showed similar findings; given this consistency, we presented only findings using BMI due to its widespread use and ease of measurement. Diabetes, hypertension, and medication use may also mediate the association. Prior studies have suggested a bidirectional association between insulin resistance and NAFLD (2), and the DiRECT trial, involving a low-calorie diet intervention, demonstrated a link between hepatic fat loss and remission of diabetes (30). Mitra and colleagues (31) postulate that NAFLD and diabetes are hallmarks of underlying metabolic dysfunction and are the hepatic and pancreatic manifestations of a systemic disorder driven by insulin resistance and inflammation. Indeed, as mentioned previously, MASLD has arisen as a new diagnostic term to refer to the combination of hepatic steatosis and metabolic dysfunction to better reflect underlying disease processes (32). Causal inference in observational cohort studies poses a challenge. However, the observed evidence supporting potential underlying biological mechanisms, combined with a long follow-up period (median of approximately 8 yr) and the consistent findings even when excluding events from the first 5 yr, strengthens the case for a temporal association between median daily step count and NAFLD risk.

In a sensitivity analysis wherein NAFLD cases were ascertained using MRI-derived PDFF measures, the association between step count and incident NAFLD was attenuated in comparison to findings in the main analysis. One possible explanation is that NAFLD ascertainment using inpatient hospital records may identify more severe cases of NAFLD, whereas NAFLD ascertainment using PDFF measures may identify a wider spectrum of NAFLD presentation, including early, asymptomatic cases. Prevention of NAFLD progression is important, as fibrosis and cirrhosis, which are only present at more advanced stages, are the strongest predictors of adverse clinical outcomes, including cardiovascular disease complications and hepatocellular carcinoma (33). Importantly, estimates were comparable using either method among the cohort of participants with both accelerometer and imaging data; this consistency recommends the validity of our observed association.

Our study has several strengths including a large sample size, objective methods for measuring physical activity, two methods for ascertaining NAFLD, small numbers of participants lost to follow-up, and comprehensive collection and consideration of confounding variables and mediating factors. In addition, although prior studies have characterized activity into relative or subjective metrics (e.g., moderate activity intensity (34) or trajectories over time (35)) our exposure variable, step count, is intuitive, accessible, and translatable beyond the context of the present study, demonstrating potential benefits for even small step count differentials. This analysis is limited by enrollment from only one geographic region with limited ethnic diversity, the possibility of inclusion of prevalent NAFLD cases, and evidence in the UK Biobank of a “healthy volunteer” bias, although previous studies in other areas suggest that many of the exposure-disease associations found in UK Biobank are largely generalizable (36). In addition, there was a time gap between assessment of physical activity and measured covariates, which may result in residual confounding. However, the calculated *E*-value for this analysis was moderately high at 1.52.

The present study demonstrates that accelerometer-measured median daily step count is robustly associated with risk of NAFLD, and this association is approximately double the strength of that reported in studies using subjective activity measurements. In addition, there is evidence that, although this association is partially mediated by adiposity, physical activity has an independent role. These findings have important implications for reducing the global impact and burden of NAFLD, a disease affecting one-third of the global population. We recognize that for some individuals, particularly those with different abilities or who face socioeconomic challenges, increasing step count may not be feasible or practical. However, we selected this exposure as it is widely understandable and, in most cases, affordable to implement. Furthermore, we would like to emphasize our finding that small changes may help lower risk (e.g., 10 more minutes of walking was associated with a 12% lower risk). Lastly, although wearable devices were used in the analysis, these findings are presented as both device-measured step count and the equivalent amount of walking time; therefore, findings are applicable to all individuals, regardless of device ownership. More research is needed to replicate these findings in other settings; however, these results advocate the importance of activity for primary prevention of NAFLD and the need for clinical trials to endorse these findings.
